# Using the Short Physical Performance Battery for Frailty Screenings Among Community-Dwelling Older Adults: An ROC Analysis

**DOI:** 10.3390/jgg74020010

**Published:** 2026-03-31

**Authors:** Eman Ali, Kworweinski Lafontant, Jethro Raphael M. Suarez, Carla Stokes Leinbach, David H. Fukuda, Jeffrey R. Stout, Sergi Garcia-Retortillo, Janet Lopez, Rui Xie, Ladda Thiamwong

**Affiliations:** 1 College of Nursing, University of Central Florida, Orlando, FL 32827, USA; 2 Institute of Exercise Physiology and Rehabilitation Science, University of Central Florida, Orlando, FL 32816, USA; 3 Department of Mechanical and Aerospace Engineering, University of Central Florida, Orlando, FL 32816, USA; 4 Disability, Aging, and Technology Cluster, University of Central Florida, Orlando, FL 32816, USA; 5 Department of Statistics and Data Science, University of Central Florida, Orlando, FL 32816, USA

**Keywords:** frailty appraisal, screen, physical frailty, low income, cross-sectional

## Abstract

Frailty is a highly prevalent and adverse syndrome among older adults, and there are many different assessments for screening both frailty (robust + pre-frail vs. frail) and frailty process (robust vs. pre-frail + frail). Previous studies have suggested that the Short Physical Performance Battery (SPPB) can be used as a quick screening tool for frailty, demonstrating excellent agreement when compared to Fried’s phenotype as a criterion. However, to the best of our knowledge, no study has assessed the SPPB’s diagnostic accuracy using the FRAIL questionnaire as the criterion. In this cross-sectional study, we compared frailty (SPPB ≤ 8) and frailty process (SPPB ≤ 10) classifications for 371 community-dwelling older adults (≥60 yrs) by the SPPB to the FRAIL questionnaire using McNemar tables and a receiver operator characteristic analysis. The SPPB and the FRAIL questionnaire significantly differed in their appraisal of both frailty and frailty process (*p* < 0.001). For frailty, the SPPB scored a sensitivity of 62.9%, a specificity of 78.6%, and an area under the curve of 0.78. In addition, for the frailty process, the SPPB scored a sensitivity of 77.6%, a specificity of 55.3%, and an area under the curve of 0.70. The SPPB demonstrated limited diagnostic accuracy compared to the criterion FRAIL questionnaire. Our findings indicate that the SPPB should not be the sole method of assessing frailty among older adults. To address the complexity of frailty, clinicians should attempt to implement multiple assessments that combine biological, social, and functional aspects of frailty. Pre-registered on ClinicalTrials.gov (NCT05778604).

## 1. Introduction

Frailty is a medical syndrome that predominantly affects older adults, and its prevalence increases with age. It is often associated with a heightened risk for falling, disability, hospitalization, and mortality [1]. Considering the significant health risks associated with frailty, it is essential to incorporate early identification and prevention strategies among at-risk older adults [2].

Currently there are over 25 different methods for screening for frailty, with few being physical assessments, and none being uniformly accepted as the measure of frailty [1]. These assessments primarily include validated questionnaires, such as the FRAIL questionnaire (fatigue, resistance, aerobic capacity, illness, and loss of weight), which are typically used for their quick screening and clinical practicality. However, these methods are not uniform and vary in their criteria, potentially leading to discrepancies in frailty classifications between methods [1], The Short Physical Performance Battery (SPPB) is a non-invasive, simple way to assess lower extremity function and mobility in older adults [3]. The SPPB test comprises three parts: a timed repeated chair stand, a timed 4-meter walk, and a 10-second balance test in a side-by-side, semi-tandem, and tandem stance [4], Additionally, the SPPB has been proposed as a potential frailty screening method [5].

A study by Perracini et al. [5] examined the SPPB as a method for measuring frailty compared to the criterion Fried’s frailty phenotype. Fried’s phenotype classifies individuals as robust, pre-frail, or frail, making it challenging for these classifications to conform to the typical binary receiver operator characteristic (ROC) analyses. Therefore, Perracini et al. [5] compared the SPPB’s frailty appraisal to the Fried’s phenotype appraisal of “frailty” (robust + pre-frail vs. frail) and “frailty process” (robust vs. pre-frail + frail). For frailty process, they observed an optimal cutoff score of ≤10, resulting in a sensitivity of 79.7%, a specificity of 73.8%, and an area under the curve (AUC) of 85%. For frailty, they observed an optimal cutoff score of ≤8, coinciding with a sensitivity of 75.5%, a specificity of 52.8%, and an AUC of 76% [5]. However, Rocco & Fernandes [6] also analyzed the diagnostic accuracy of the SPPB compared to Fried’s phenotype, noting an optimal cutoff of ≤6 (sensitivity = 28.0%; specificity = 94.0%; and AUC = 61%) despite using a similar sample of Brazilian older adults. These equivocal results highlight the need for further research on the diagnostic accuracy of the SPPB for appraising frailty among older adults, especially given its popular clinical use [7,8].

To the best of our knowledge, no study has evaluated the diagnostic accuracy of the SPPB compared to the FRAIL Questionnaire. The FRAIL questionnaire is based on the same five criteria as Fried’s phenotype and was designed to provide a faster and more clinically practical frailty assessment compared to Fried’s phenotype without the need for equipment such as a handgrip dynamometer [7,9]. Similarly, the SPPB is an accessible test that requires minimal equipment and provides insight into physical functioning [7,8], Given the heightened risk of frailty among older adults, clinicians require a quick and straightforward method to assess frailty in older adults, and both the SPPB and FRAIL questionnaire have the potential to fill that gap. While the FRAIL questionnaire may be viewed as a faster alternative that older adults can complete themselves to self-screen for frailty, many clinicians may still elect to use the SPPB to screen for frailty, in line with previous research demonstrating a desire by older patients for more face-to-face care and clinician-patient interactions [10,11], However, the SPPB does not directly address the five domains of frailty proposed by Fried [12] and therefore cannot function as a criterion measure of frailty. Moreover, it is currently unclear how the SPPB’s assessment of frailty compares to the FRAIL questionnaire, as Perracini et al. [5] only used Fried’s frailty phenotype as a criterion. Therefore, the purpose of this study was to conceptually replicate the work of Perracini et al. [5] by evaluating the diagnostic accuracy of the SPPB in identifying frailty among community-dwelling older adults when compared to the FRAIL questionnaire. This type of replication is designed to determine if the underlying concept of using a physical function test to screen for frailty in place of the five frailty domains (addressed by both the FRAIL questionnaire and Fried’s frailty phenotype) functions similarly in our work as it did with Perracini et al. [5,13]. We hypothesized that the SPPB would achieve a sensitivity of at least 25.0% and a specificity of at least 80.0% in line with previous research [6].

## 2. Materials and Methods

### 2.1. Study Design

This was a secondary cross-sectional analysis of a larger clinical trial (ClinicalTrials.gov, NCT05778604), the methods of which are published elsewhere [14]. The study methods were approved by the University of Central Florida Institutional Review Board (STUDY00003206, approved 8 September 2021) and were done in accordance with the Declaration of Helsinki. All the participants provided written informed consent prior to participation in the study. Between February 2023 and August 2025, we recruited a total of 404 low-income community-dwelling older adults via word of mouth as well as flyers and pamphlets posted in community centers, health fairs, and senior living centers all within the greater Orlando, Florida, region. Additionally, participants received a $50 gift card after completion of the study visit. Participants were included in the study if they were (i) ≥60 years old, (ii) had low-income status, based on 2019 U.S. Census guidelines [15], and (iii) were able to perform all three elements of the SPPB.

### 2.2. Frailty Assessment

Frailty was assessed using the FRAIL questionnaire, which evaluates fatigue, resistance, ambulation, illness, and loss of weight [9], The questionnaire is a quick and simple method commonly used in assessing frailty, and the tool has been validated for use among diverse populations [9,16]. Each participant completed the questionnaire, in person and on paper, receiving a total score ranging from 0 to 5. Based on their overall score, they were categorized as robust (score = 0), pre-frail (score = 1–2), or frail (score = 3–5). In line with the definitions established by previous research for ROC analyses [5,9,16], the robust and pre-frail groups (score = 0–2) were combined and compared to frail (score = 3–5) when assessing “frailty.” When assessing “frailty process,” the pre-frail and frail groups (score = 1–5) were combined and compared to the robust group (score = 0) [5,9,16].

### 2.3. Short Physical Performance Battery

The SPPB involves three sub-tests for the assessment of physical function, namely, standing balance, usual gait speed, and repeated chair stands, in that order [4]. To standardize the instructions, timers used, and scoring, the SPPB was administered by trained research assistants utilizing an SPPB Guide phone application [17], For the balance component, participants were instructed to stand unassisted in three different positions (feet side-by-side, semi-tandem, and full tandem) for 10 s each. In the assessment of gait speed, participants walked four meters at their usual walking speed for two trials, and the times for each trial were recorded and averaged together. Participants were then asked to complete five sit-to-stand repetitions as fast as possible, beginning in the seated position with their arms crossed on their chest and both feet flat on the floor. Each of the categorical scores ranged from 0 to 4 based on standard scoring guides detailed elsewhere [4], producing a total composite score ranging from 0 to 12.

### 2.4. Statistical Analysis

The data used for the study was stored on a Research Electronic Database Capture (REDCap) database managed by the University of Central Florida [18,19], All statistical analyses were performed using jamovi version 2.5.6 and the *Psychometrics & Post-Data Analysis* module within jamovi [20–23], McNemar tables were used to compare frailty classifications between the SPPB and FRAIL questionnaires for both frailty and frailty process. We conducted an ROC analysis for continuous SPPB scores using the FRAIL questionnaire as the criterion, adopting the frailty and frailty process groupings and cutoff scores from previous research [5,20] to determine sensitivity (True Positives/[True Positives + False Negatives]), specificity (True Negatives/[True Negatives + False Positives]), accuracy ([True Positives + True Negatives]/N), positive predictive value (PPV; [True positives/(True Positives + False Positives)]), negative predictive value (NPV; [True Negatives/(True Negatives + False Negatives)]), and AUC. While Perracini et al. [5] provided cutoff values, we also identified optimal cutoff scores within our dataset via the greatest observed Youden’s Index (j). The threshold for statistical significance was set at *p* < 0.05. All data are presented as mean ± standard deviation unless otherwise indicated.

## 3. Results

### 3.1. Participants

**Figure 1.**
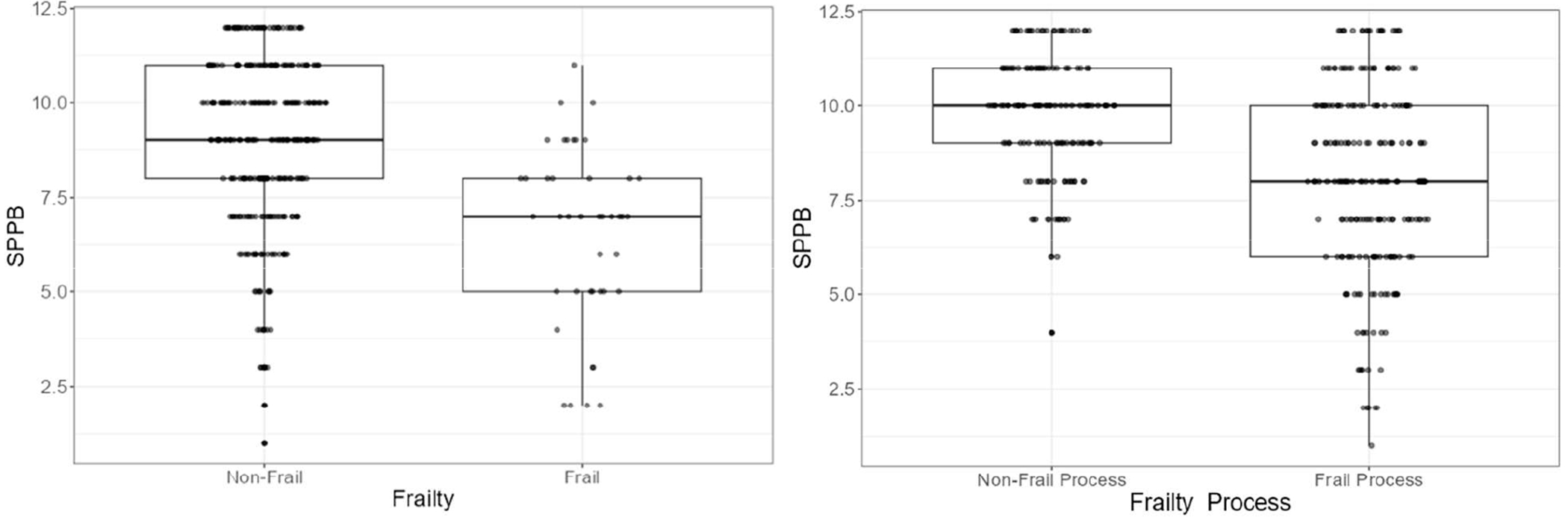
Distribution of Short Physical Performance Battery (SPPB) scores using frailty and frailty process classifications from the FRAIL questionnaire (*N* = 371). With the frailty classification, 42 participants were classified as frail and 329 were classified as non-frail. With the frailty process classification, 219 participants were classified as frail and 152 were classified as non-frail.

From the 404 recruited low-income community-dwelling older adults, 371 (female, *n* = 325; male, *n* = 46) participants met the inclusion criteria and were analyzed in this study. Table 1 provides the demographic information for all analyzed participants. Figure 1 shows the distribution of SPPB scores based on frailty (frail = 6.57 ± 2.36; non-frail = 9.02 ± 2.22) and frailty process classifications (frail = 8.04 ± 2.53; non-frail = 9.74 ± 1.65).

### 3.2. Frailty Process Classification

**Figure 2.**
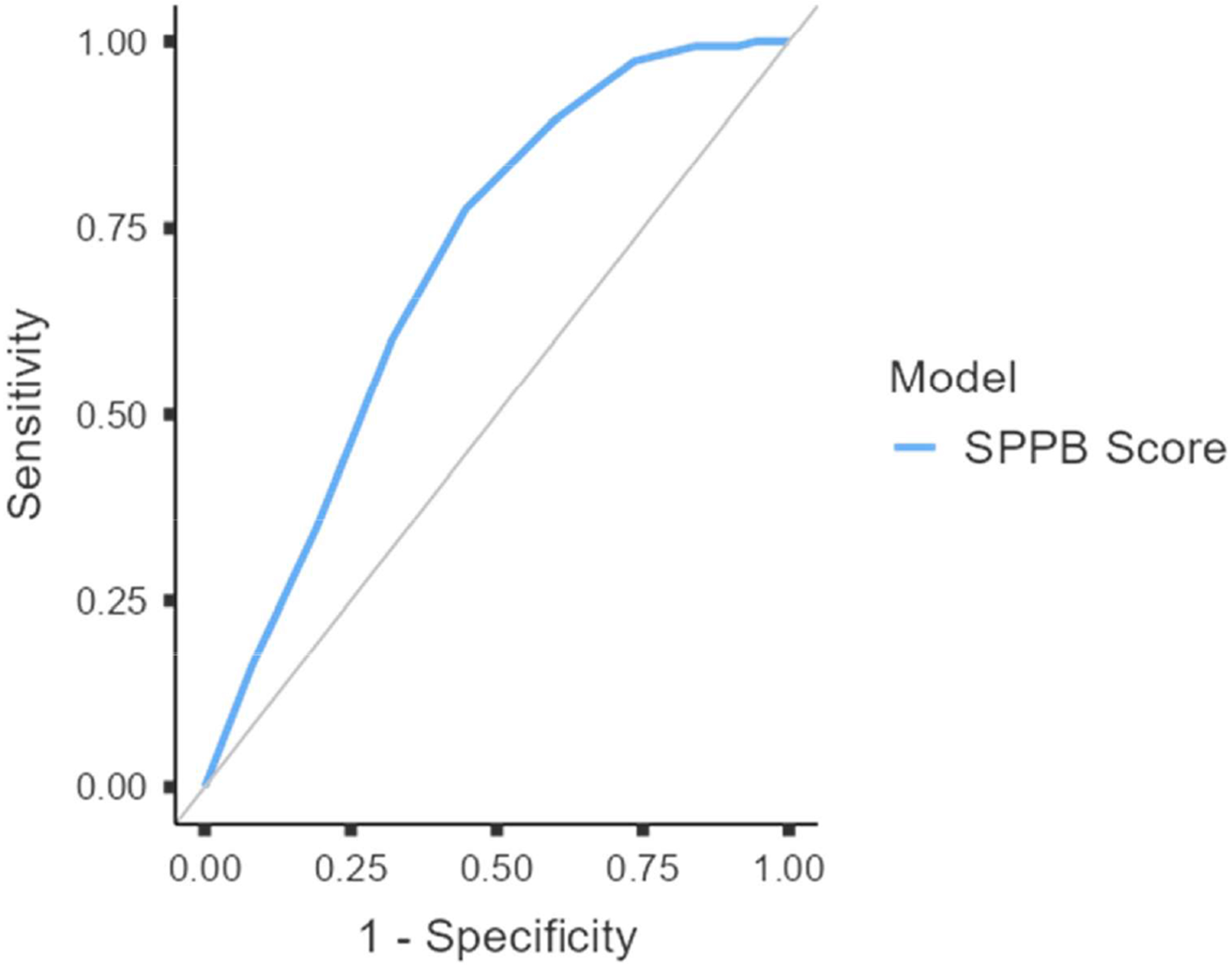
Receiver operator characteristic curve for frailty process (i.e., frail + pre-frail vs. robust) comparing SPPB scores (i.e., the blue curved line) with FRAIL questionnaire scores as the criterion.

Table 2 shows a cross analysis of the SPPB with a cutoff score ≤ 10, based on Perracini et al. [5], and the FRAIL questionnaire scores of the participants for frailty process. Figure 2 shows the observed AUC for the SPPB in assessing frailty process. Compared to the FRAIL questionnaire as the criterion, we observed a sensitivity of 77.6%, a specificity of 55.3%, an accuracy of 64.4%, an AUC of 70%, a PPV of 54.6%, and an NPV of 78.1%. Based on Youden’s Index (j = 0.33), we observed an optimal SPPB cutoff score of 8.5 (i.e., ≤8) for frailty process. From the McNemar test, the SPPB and the FRAIL questionnaire significantly differed in their appraisal of frailty process when using Perracini et al.’s [5] cutoff score of 10 (X^2^ = 23.0; *p* < 0.001). When using the observed cutoff score of 8.5 from the present study, significant differences in frailty process appraisal remained (X^2^ = 31.0; *p* < 0.001).

### 3.3. Frailty Classification

**Figure 3.**
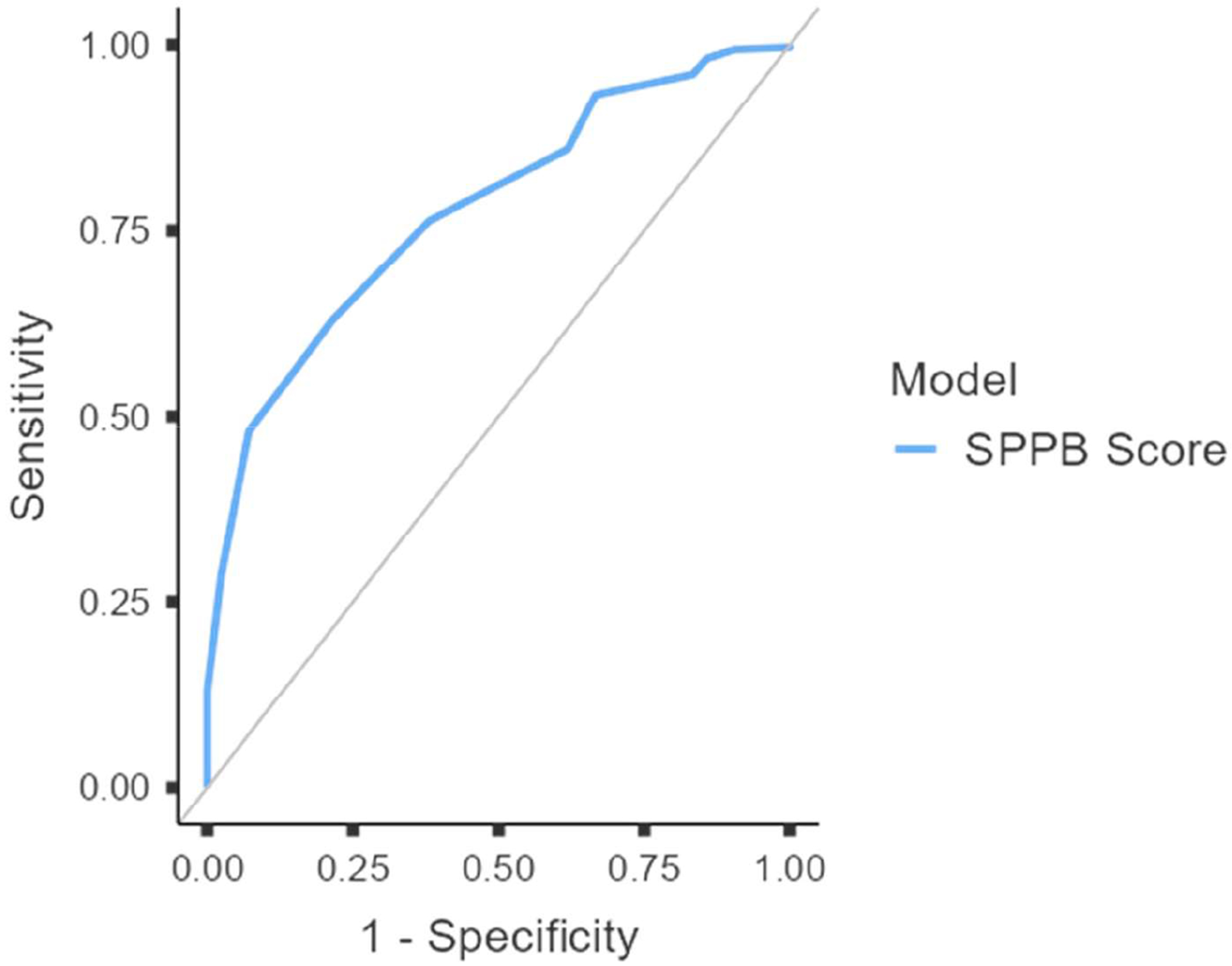
Receiver operator characteristic curve for frailty (i.e., frail vs. pre-frail + robust) comparing SPPB scores (i.e., the blue curved line) with FRAIL questionnaire scores as the criterion.

Table 3 shows a cross-analysis of the SPPB with a cutoff score ≤8 and the FRAIL questionnaire scores of the participants for frailty. Figure 3 shows the AUC for the SPPB in assessing frailty. Compared to the FRAIL questionnaire as the criterion, with an SPPB cutoff score of 8.5 (i.e., ≤8), we observed a sensitivity of 62.9%, a specificity of 78.6%, an accuracy of 64.7%, an AUC of 78%, a PPV of 95.8%, and an NPV of 21.3%. Similar to Perracini et al. [5], we observed an optimal cutoff score of 8.5 (i.e., <8), based on Youden’s Index (j = 0.41). From the McNemar test, the SPPB and the FRAIL questionnaire significantly differed in their appraisal of frailty (X^2^ = 97.5; *p* < 0.001).

## 4. Discussion

The purpose of this study was to assess the validity of the SPPB in screening for frailty among community-dwelling older adults when compared to the FRAIL questionnaire. Our initial hypothesis that the SPPB would reach a sensitivity and specificity of at least 80.0% differed from the results obtained. For the frailty process with an observed cutoff of scores ≤ 8, the SPPB scored a sensitivity of 77.6%, a specificity of 55.3%, and an AUC of 70.0%. Additionally, for frailty (SPPB ≤ 8), the SPPB scored a sensitivity of 62.9%, a specificity of 78.6%, and an AUC of 78.0%. While the observed AUC values were greater than 0.50, indicating greater diagnostic accuracy than random chance (i.e., 50%), the observed AUCs were moderate at best. These results suggest that employing the SPPB as a screening tool for frailty and frailty process via the FRAIL questionnaire may not provide the most accurate results.

The present results were somewhat similar to those of Perracini et al. [5] as they observed a sensitivity of 79.7% and a specificity of 73.8% for frailty, while for frailty process, they saw a sensitivity of 75.5% and a specificity of 52.8%. However, it is important to note that Perracini et al. [5] utilized Fried’s phenotype rather than the FRAIL questionnaire to determine frailty. A key difference between the tools is that, despite both assessments focusing on the same domains of frailty, the FRAIL questionnaire does not include any objective physical assessments while Fried’s phenotype includes physical tests for gait speed and handgrip strength [12]. Previous research has suggested that older adults may over- or underestimate their physical activity levels when self-reporting compared to objective measures [24], suggesting that similar discrepancies may exist when self-reporting physical functionality. Given that the FRAIL questionnaire relies on self-reporting, it is plausible that the FRAIL questionnaire and Fried’s phenotype differ as criterion measures for frailty, leading to the slight differences observed between the present study and Perracini et al. [5] regarding the SPPB.

The SPPB was designed to be a robust objective assessment of physical function [4], which may explain its slightly stronger relationship with Fried’s phenotype than the FRAIL questionnaire, as Fried’s phenotype utilized physical assessments. However, the SPPB’s sole focus on physical function may limit its capability in determining frailty, as frailty is complex in nature. Beyond manifestations in physical function, frailty is often characterized by depressive symptoms, cognitive disorders, and a lack of social support [25], Self-reported feelings of exhaustion are included in both Fried’s phenotype and the FRAIL questionnaire as a key component of frailty [9,12], allowing both tools to somewhat assess emotional aspects of frailty. Yet, the SPPB does not include any provision to assess frailty beyond its physical function manifestations, which may limit its clinical utility. To assess all aspects of frailty, a screening tool must include questions and / or tasks that address the physical, emotional, social, and cognitive aspects of frailty, among others. Given the complex nature of frailty, there may be a need for future research to develop an algorithmic approach to frailty screening within clinical practice, as one single screening test may not adequately address all aspects of frailty.

While this study was strengthened by its large sample size, diverse population, and non-invasive and clinically practical methods, several limitations must be acknowledged. Almost 90% of our participants were female, which limits our ability to confidently generalize our results to male older adults. This may have been due to our recruitment methods, which were largely focused on community spaces for older adults, as previous research has indicated a global trend towards decreased community-based social engagement among older men [26–28]. However, it is worth noting that many frailty assessments, including Fried’s phenotype, the FRAIL questionnaire, and the SPPB, do not differentiate between men and women in their frailty appraisal criteria [1], indicating a need to assess the validity of these tools with a mixed-sex sample. Future research should aim to replicate our work using a targeted recruitment strategy for older men. Our sample was also limited to low-income community-dwelling older adults as part of the larger study’s focus, and this may impact the ability to translate our observations to older adults with higher socioeconomic status. Lower socioeconomic status is understood to be associated with a higher prevalence of frailty and potentially of non-physical aspects of frailty (e.g., social, environmental, etc.) [29,30], which may not be adequately captured by the SPPB. Further research is needed with mixed socioeconomic status samples to explore potential mediating effects on diagnostic accuracy when screening frailty. Additionally our use of a cross-sectional analysis limited our ability to determine prospective accuracy of the SPPB. Future studies should employ a longitudinal design to determine if the cross-sectional accuracy of various frailty assessments relates to frailty incidence over time. Given that there are over 20 different methods for assessing frailty and no consensus on a criterion method [31,32], further research is needed to determine how the frailty appraisals between different screening methods compare. This is especially necessary as more novel frailty assessments are being developed with advanced objective techniques beyond those used within Fried’s phenotype [33].

## 5. Conclusions

The SPPB demonstrated limited diagnostic accuracy when assessing frailty and frailty process compared to the FRAIL questionnaire. This suggests that the SPPB should not be the only tool used to screen for frailty in older adults. Clinicians should consider combining the SPPB with other diagnostic tests (e.g., FRAIL questionnaire, Fried’s phenotype, etc.) to capture potential social, mental, and emotional aspects of frailty and improve overall screening accuracy.

## Figures and Tables

**Table 1.** Participant demographics.

	All (*N* = 371)	Women (*n* = 325)	Men (*n* = 46)
Age (years)	74.1 ± 8.04	74.1 ± 8.15	74.2 ± 7.31
BMI (kg/m^2^)	30.1 ± 6.37	30.3 ± 6.21	28.8 ± 7.32
Body Mass (kg)	77.2 ± 18.1	76.3 ± 17.5	83.0 ± 21.5
	AA: 177 (47.7%)	AA: 163 (50.0%)	AA: 14 (30.4%)
	A: 24 (6.5%)	A: 18 (5.5%)	A: 6 (13.0%)
Race/Ethnicity	H: 93 (25.1%)	H: 84 (25.9%)	H: 9 (19.6%)
	NHW: 74 (19.9%)	NHW: 57 (17.6%)	NHW: 17 (37.0%)
	WI: 3 (0.8%)	WI: 3 (1.0%)	WI: 0 (0.0%)

Note. AA = African American; A = Asian; H = Hispanic; NHW = Non-Hispanic White; WI = West Indian; BMI = Body Mass Index. Data are presented as mean ± standard deviation or *n* (%).

**Table 2.** Cross-tabulation of SPPB and FRAIL questionnaire for frailty process.

Cutoff Score: ≥10	FRAIL Frailty Process	FRAIL Non-Frail Process	Total
SPPB—Frail Process	177	99	276
SPPB—Non-Frail Process	42	53	95
Total	219	152	371
Cutoff Score: ≤8	FRAIL Frailty Process	FRAIL Non-Frail Process	Total

SPPB—Frail Process	121	34	155
SPPB—Non-Frail Process	98	118	216
Total	219	152	371

Note. The cutoff score of ≤10 for the Short Physical Performance Battery (SPPB) was based on Perracini et al. [5], while the cutoff score of 8.5 was from the present study. Frailty process was determined by the FRAIL questionnaire by separating the robust group from the combined pre-frail and frail groups. Each number represents the given group or subgroup’s sample size (*n*).

**Table 3.** Cross-tabulation of SPPB and FRAIL questionnaire for frailty.

Cutoff Score: ≤8	FRAIL Frail	FRAIL Non-Frail	Total
SPPB—Frail	33	122	155
SPPB—Non-Frail	9	207	216
Total	42	329	371

Note. The cutoff score of ≤8 (i.e., cutoff of 8.5) was used for the Short Physical Performance Battery (SPPB) based on both Perracini et al. [5] and data from the present study. Frailty was determined by the FRAIL questionnaire by separating the combined robust and pre-frail groups from the frail group. Each number represents the given group or subgroup’s sample (*n*).

## Data Availability

The data presented in this study are available on request from the corresponding author due to ethical reasons (public posting of data into a repository was not specified a priori within informed consent for participants).

## Abbreviations

The following abbreviations are used in this manuscript:

SPPB: Short Physical Performance Battery
FRAIL: Fatigue, resistance, ambulation, illness, loss of weight
ROC: Receiver operator curve
AUC: Area under the curve
PPV: Positive predictive value
NPV: Negative predictive value

## References

[1] Pritchard JM; Kennedy CC; Karampatos S; loannidis G; Misiaszek B; Marr S; Patterson C; Woo T; Papaioannou A Measuring frailty in clinical practice: A comparison of physical frailty assessment methods in a geriatric out-patient clinic. BMC Geriatr. 2017,17, 264. 29132301 10.1186/s12877-017-0623-0 PMC5683585

[2] Gonçalves R.S.d.S.A.; Ribeiro K.M.O.B.d.F.; Fernandes SGG; de Andrade LEL; Lira M.d.G.d.A.; do Nascimento RA; Vieira MCA; Maciel ÁCC Diagnostic Accuracy of the Short Physical Performance Battery in Detecting Frailty and Prefrailty in Community-Dwelling Older Adults: Results From the PRO-EVA Study. J. Geriatr. Phys. Ther 2023, 46, E127–E136. 35470303 10.1519/JPT.0000000000000352

[3] Bergland A; Strand BH Norwegian reference values for the Short Physical Performance Battery (SPPB): The Tromsø Study. BMC Geriatr. 2019,19, 216. 31395008 10.1186/s12877-019-1234-8 PMC6686475

[4] Guralnik JM; Simonsick EM; Ferrucci L; Glynn RJ; Berkman LF; Blazer DG; Scherr PA; Wallace RB A short physical performance battery assessing lower extremity function: Association with self-reported disability and prediction of mortality and nursing home admission. J. Gerontol 1994, 49, M85–M94. 8126356 10.1093/geronj/49.2.m85

[5] Perracini MR; Mello M; de Oliveira Máximo R; Bilton TL; Ferriolli E; Lustosa LP; da Silva Alexandre T Diagnostic Accuracy of the Short Physical Performance Battery for Detecting Frailty in Older People. Phys. Ther 2019,100, 90–98. 10.1093/ptj/pzz154 31612228

[6] Rocco LLG; Fernandes TG Validity of the short physical performance battery for screening for frailty syndrome among older people in the Brazilian Amazon region. A cross-sectional study. Sào Paulo Med. J 2020,138, 537–544. 33263707 10.1590/1516-3180.2020.0264.R1.14092020 PMC9685569

[7] Banarjee C; Choudhury R; Park J-H; Xie R; Fukuda D; Stout J; Thiamwong L Common Physical Performance Tests for Evaluating Health in Older Adults: Cross-Sectional Study. Interact. J. Med. Res 2024, 13, e53304. 39612490 10.2196/53304 PMC11645506

[8] Welch SA; Ward RE; Beauchamp MK; Leveille SG; Travison T; Bean JF The Short Physical Performance Battery (SPPB): A Quick and Useful Tool for Fall Risk Stratification Among Older Primary Care Patients. J. Am. Med. Dir. Assoc 2021, 22,1646–1651. 33191134 10.1016/j.jamda.2020.09.038 PMC8113335

[9] Gleason LJ; Benton EA; Alvarez-Nebreda ML; Weaver MJ; Harris MB; Javedan H FRAIL Questionnaire Screening Tool and Short-Term Outcomes in Geriatric Fracture Patients. J. Am. Med. Dir. Assoc 2017,18,1082–1086. 28866353 10.1016/j.jamda.2017.07.005 PMC6611671

[10] Hobden B; Mansfield E; Freund M; Clapham M; Sanson-Fisher R Experiences of patient-centered care among older community-dwelling Australians. Front. Public Health 2022,10, 912137. 35774564 10.3389/fpubh.2022.912137 PMC9237321

[11] Williams SL; Haskard KB; DiMatteo MR The therapeutic effects of the physician-older patient relationship: Effective communication with vulnerable older patients. Clin. Interv. Aging 2007, 2, 453–467. 18044195 PMC2685265

[12] Fried LP; Tangen CM; Walston J; Newman AB; Hirsch C; Gottdiener J; Seeman T; Tracy R; Kop WJ; Burke G Frailty in older adults: Evidence for a phenotype.J. Gerontol. Ser. A Biol. Sei. Med. Sei 2001, 56, M146–M157. 10.1093/gerona/56.3.m146 11253156

[13] Derksen M; Morawski J Kinds of replication: Examining the meanings of “conceptual replication” and “direct replication”. Perspect. Psychol. Sci 2022,17,1490–1505. 35245130 10.1177/17456916211041116 PMC9442273

[14] Thiamwong L; Xie R; Park JH; Lighthall N; Loerzel V; Stout J Optimizing a Technology-Based Body and Mind Intervention to Prevent Falls and Reduce Health Disparities in Low-Income Populations: Protocol for a Clustered Randomized Controlled Trial. JMIR Res. Protoc 2023,12, e51899. 37788049 10.2196/51899 PMC10582821

[15] Historical Poverty Thresholds. Available online: https://www.census.gov/data/tables/time-series/demo/income-poverty/historical-poverty-thresholds.html (accessed on 8 September 2021).

[16] Morley JE; Malmstrom TK; Miller DK A simple frailty questionnaire (FRAIL) predicts outcomes in middle aged African Americans. J. Nutr. Health Aging 2012,16, 601–608. 22836700 10.1007/s12603-012-0084-2 PMC4515112

[17] Corporation, N.P. SPPB Guide. 2021. Available online: https://www.sppbguide.com (accessed on 8 September 2021).

[18] Harris PA; Taylor R; Minor BL; Elliott V; Fernandez M; O’Neal L; McLeod L; Delacqua G; Delacqua F; Kirby J; The REDCap consortium: Building an international community of software platform partners. J. Biomed. Inform 2019, 95,103208. 31078660 10.1016/j.jbi.2019.103208 PMC7254481

[19] Harris PA; Taylor R; Thielke R; Payne J; Gonzalez N; Conde JG Research electronic data capture (REDCap)—A metadata-driven methodology and workflow process for providing translational research informatics support. J. Biomed. Inform 2009, 42, 377–381. 18929686 10.1016/j.jbi.2008.08.010 PMC2700030

[20] Thiele C Determine and Evaluate Optimal Cutpoints in Binary Classifcation Tasks. 2018. Available online: https://cran.r-project.org/web/packages/cutpointr/cutpointr.pdf (accessed on 9 January 2026).

[21] R: A Language and Environmental for Statistical Computing; R Foundation for Statistical Computing: Vienna, Austria, 2022.

[22] Love J; Dropmann D; Selker R; Gallucci M; Jentschke S; Balci S; Seol H; Agosti M jamovi, version 2.5.6; jamovi project: Sydney, Australia, 2024.

[23] Friesen L; Kroc E; Zumbo BD Psychometrics & Post-Data Analysis: Test ROC; GitHub: San Francisco, CA, USA, 2019.

[24] VandeBunte A; Gontrum E; Goldberger L; Fonseca C; Djukic N; You M; Kramer JH; Casaletto KB Physical activity measurement in older adults: Wearables versus self-report. Front. Digit. Health 2022, 4, 869790. 36120711 10.3389/fdgth.2022.869790 PMC9470756

[25] Wleklik M; Uchmanowicz I; Jankowska EA; Vitale C; Lisiak M; Drozd M; Pobrotyn P; Tkaczyszyn M; Lee C Multidimensional approach to frailty. Front. Psychol 2020, 11 564. 32273868 10.3389/fpsyg.2020.00564 PMC7115252

[26] Davidson K; Daly T; Arber S Older Men, Social Integration and Organisational Activities. Soc. Policy Soc. A J. Soc. Policy Assoc 2003, 2, 81–89.

[27] Thompson EH; Whearty PM Older Men’s Social Participation: The Importance of Masculinity Ideology. J. Men’s Stud 2004,13, 5–24.

[28] Naud D; Généreux M; Bruneau J-E; Alauzet A; Levasseur M Social participation in older women and men: Differences in community activities and barriers according to region and population size in Canada. BMC Public Health 2019, 19, 1124. 31420061 10.1186/s12889-019-7462-1 PMC6697934

[29] Mangin D; Lawson J; Risdon C; Siu HY-H; Packer T; Wong ST; Howard M Association between frailty, chronic conditions and socioeconomic status in community-dwelling older adults attending primary care: A cross-sectional study using practice-based research network data. BMJ Open 2023, 13, e066269. 10.1136/bmjopen-2022-066269 PMC9944661 36810183

[30] Hanlon P; Politis M; Wightman H; Kirkpatrick S; Jones C; Khan M; Bezzina C; Mackinnon S; Rennison H; Wei L Frailty and socioeconomic position: A systematic review of observational studies. Ageing Res. Rev 2024,100,102420. 39025269 10.1016/j.arr.2024.102420

[31] Gary R Evaluation of frailty in older adults with cardiovascular disease: Incorporating physical performance measures. J. Cardiovasc. Nurs 2012, 27,120–131. 22334147 10.1097/JCN.0b013e318239f4a4 PMC3664396

[32] Lafontant K; Fukuda DH; Zamarripa E; Tice AL; Suarez JRM; Banarjee C; Kim D; Stout JR; Park J-H; Xie R Application of Levi’s Muscle Index in frailty assessment: Comparison of bioimpedance measures among older adults. Front. Med 2025,12,1525569. 10.3389/fmed.2025.1525569 PMC12243873 40641985

[33] Newman AB; Blackwell TL; Mau T; Cawthon PM; Coen PM; Cummings SR; Toledo FGS; Goodpaster BH; Glynn NW; Hepple RT; Vigor to Frailty As a Continuum—A New Approach in the Study of Muscle, Mobility, and Aging Cohort. J. Gerontol. Ser. A 2023, 79, glad244. 10.1093/gerona/glad244 PMC10733210 37847228

